# Mapping the neuroimmune landscape of Alzheimer’s disease in African Americans

**DOI:** 10.1002/alz.091633

**Published:** 2025-01-03

**Authors:** Nkechime E. Ifediora, Nivedita Nimmagadda, Emily Lee, Peter D. Canoll, Julie A. Schneider, David A. Bennett, Vilas Menon, Lisa L. Barnes, Marta Olah

**Affiliations:** ^1^ Columbia University Irving Medical Center, New York, NY USA; ^2^ Rush Alzheimer’s Disease Center, Rush University Medical Center, Chicago, IL USA; ^3^ The Taub Institute for Research on Alzheimer’s Disease and The Aging Brain, New York, NY USA; ^4^ Center for Translational & Computational Neuroimmunology, New York, NY USA

## Abstract

**Background:**

African Americans (AA) are disproportionally burdened by Alzheimer’s disease (AD), but there is a scarcity of research focusing on understanding the neuroimmune component of AD pathogenesis in this population. It is generally accepted that microglia would be an ideal therapeutic target for AD and that genetic, lifestyle, societal and environmental factors and stressors have the potential to shape microglia phenotypes and their contribution to neurodegenerative processes. The overarching goal of the current study is to establish the population structure of microglia in older AAs and to investigate the relationship of the different microglia subsets with histopathological hallmarks of brain aging and AD in AAs.

**Method:**

We utilized our established pipeline to isolate live microglia (and other immune cells) from human brain and investigate them at the single cell level using a multi‐omic approach employing transcriptomic, epigenetic and proteomic profiling. This approach, that has recently yielded the first map of microglia population structure in the aged human brain of NHW donors, has been supplemented with in situ investigation of microglia phenotypes and their association with AD pathology in archival tissue specimens.

**Result:**

We isolated and profiled live microglia from the dorsolateral prefrontal cortex and the anterior watershed deep white matter of 10 AA donors from the Minority Aging Research Study (MARS). Both of these brain regions have been previously associated with cognitive decline. Using single cell RNA‐sequencing, unsupervised hierarchical clustering of the data revealed nine distinct microglia phenotypes: two homeostatic subsets and seven differentiated subsets, similarly to what we previously described in NHW donors. Intriguingly, we detected a different population structure of microglia in AA donors when compared to NHWs. To establish an in depth understanding of these findings we investigated the chromatin accessibility and proteomic profile of the different subsets as well as their relationship to histopathological traits of brain aging in AA donors.

**Conclusion:**

While microglia in the cortex and white matter of older AA donors acquire the same functional phenotypes as microglia in the brains of NHW donors, they display a distinct population structure. Our findings underline the importance of studying the neuroimmune compartment of AD specifically in AAs.

